# Estimating tumour immune infiltration: methodological convergence across histology and spatial technologies

**DOI:** 10.1093/bib/bbag176

**Published:** 2026-05-15

**Authors:** Beilei Bian, Yue Cao, Jean Yee Hwa Yang

**Affiliations:** School of Mathematics and Statistics, The University of Sydney, F07 Eastern Avenue, Sydney, Australia; Sydney Precision Data Science Centre, University of Sydney, F07 Eastern Avenue, Sydney, Australia; Charles Perkins Centre, The University of Sydney, Johns Hopkins Drive, Sydney, Australia; School of Mathematics and Statistics, The University of Sydney, F07 Eastern Avenue, Sydney, Australia; Sydney Precision Data Science Centre, University of Sydney, F07 Eastern Avenue, Sydney, Australia; Charles Perkins Centre, The University of Sydney, Johns Hopkins Drive, Sydney, Australia; School of Mathematics and Statistics, The University of Sydney, F07 Eastern Avenue, Sydney, Australia; Sydney Precision Data Science Centre, University of Sydney, F07 Eastern Avenue, Sydney, Australia; Charles Perkins Centre, The University of Sydney, Johns Hopkins Drive, Sydney, Australia

**Keywords:** tumour immune infiltration, histology, spatial omics, computational methods, multimodal data integration

## Abstract

Estimating tumour immune infiltration is critical for understanding cancer biology and predicting patient response to surgery and immunotherapy. A wide array of experimental platforms supports various computational approaches for quantifying tumour-infiltrating lymphocytes and immune infiltration level within the tumour microenvironment, including traditional histopathology, immunohistochemistry, AI-based digital pathology, bulk RNA sequencing, and spatial omics now. Although numerous technologies are available to quantify immune infiltration, important questions remain about which approaches are most suitable and how best to guide platform and methodological choices. In this review, we provide a comprehensive overview of how each platform estimates tumour immune profile coupled with their corresponding computational approaches, followed by a comparative discussion on technological resolution, cell-type specificity, spatial context, and clinical interpretability. We also discuss emerging trends in multimodal data integration, including mapping-based and fusion-based strategies. Together, our review underscores both the methodological opportunities and the translational potential of diverse immune infiltration estimation strategies, guiding the design of more actionable immune profiling strategies.

## Introduction

Tumour-infiltrating lymphocytes (TILs) are white blood cells (including T cells and B cells) that move from blood into tumours and work to kill cancer cells as cancers grow. TILs play an important role in cancer biology and has been identified as a key prognostic factor beyond traditional tumour staging and molecular characteristics, with aggregated and highly dense TILs favouring better clinical outcomes [1–4]. In addition to being a biomarker of cancer progression and responses to treatment, TILs can also be used as a cellular treatment for various cancers. TIL therapy was first proposed by Rosenberg and colleagues in 1986 using a mouse model [5] and approved by The Food and Drug Administration (FDA) for treating advanced melanoma more recently [6].

Despite its clinical importance, accurately assessing TILs in tumours remains a major challenge. Tumours are inherently heterogeneous, not only between patients but also within different regions of the same tumour, leading to variable patterns of immune cell distribution [7, 8]. Classical histological methods, such as hematoxylin and eosin (H&E) staining and immunohistochemistry (IHC), provide valuable morphological and cell type information but rely heavily on subjective interpretation by pathologists. This manual assessment introduces inter-observer variability and limits the scalability of immune profiling [9]. Furthermore, conventional techniques often lack fine-grained cell subtype information and the sensitivity to capture subtle spatial and functional nuances of the immune microenvironment [10], highlighting the need for advanced technologies and more robust, quantitative, and reproducible analytical approaches.

**Figure 1:**
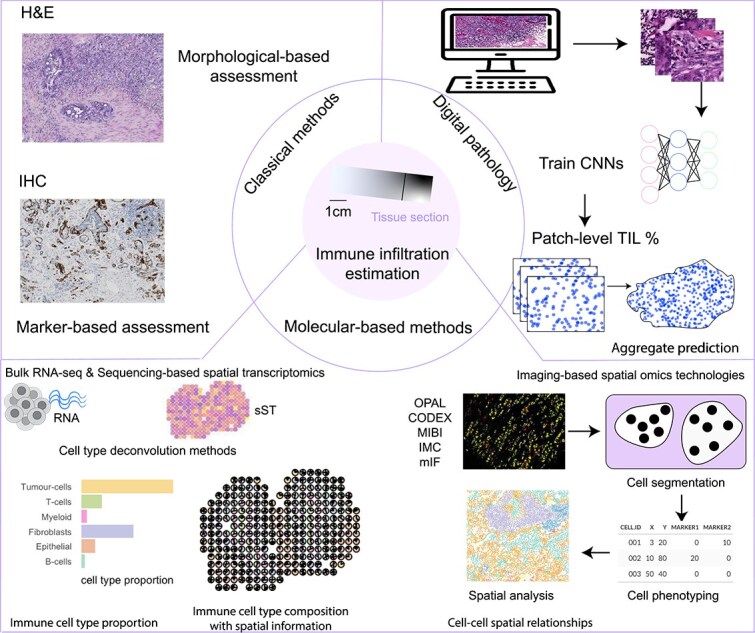
Multiple platforms for estimating immune infiltration. Multiple experimental platforms and computational methods have been developed for assessing TILs and broader immune infiltration. Classical pathology-based methods include morphological assessment of TILs on H&E-stained sections by pathologists and marker-based assessment using IHC to detect immune cell subsets. Digital pathology approaches leverage machine learning and computer vision, where CNNs are trained on histology images to predict patch-level TIL percentages and generate aggregate predictions across whole slides. Molecular-based methods include bulk RNA-sequencing and sST, combined with cell type deconvolution to estimate immune cell proportions with or without spatial resolution. Imaging-based spatial omics technologies (e.g. OPAL, CODEX, MIBI, IMC, and multiplexed immunofluorescence) enable high-dimensional mapping of immune and stromal cell populations. These platforms rely on cell segmentation and phenotyping, and facilitate spatial analyses of cell-cell interactions within the tumor microenvironment. Together, these complementary methods provide quantitative and spatially resolved estimates of immune infiltration in cancer tissues.

In recent years, a range of advanced technologies and computational tools have emerged to enable the field to address these limitations, advancing our understanding of the tumour immune microenvironment in a more computationally and molecularly driven manner (Fig. 1). For example, advances in digital pathology have enabled the use of AI models to automatically quantify TILs from H&E images, enhancing consistency and scalability compared to manual assessment [11–13]. In parallel, molecular-based technologies (bulk RNA-seq, single-cell RNA-seq, sequencing-based spatial transcriptomics (sST), and imaging-based spatial omics) coupled with tailored computational methods, provide opportunities to characterize immune cell subtypes and spatial distributions at varying resolutions, offering complementary insights beyond classical H&E and IHC.

The growing computational frameworks for multimodal data integration are enabling complementary use of diverse data types, offering new opportunities to capture the complexity of tumour immune microenvironment. These data integration approaches can uncover cross-modal patterns that cannot be detected using a single platform, which hold promise for comprehensively characterizing immune infiltration.

In this review, we first briefly summarize statistical measures and approaches for estimating the immune infiltration level or TILs under each platform. We further highlight the growing trend of multimodal data integration using deep learning frameworks. We then present a comparative discussion on the strengths and limitations for TILs estimation using each platform from different perspectives. This offers opportunities for guiding researchers to choose appropriate platforms based on specific research questions, resolution requirements, and desired applicability.

## Classical histology-based estimation

### Hematoxylin and eosin staining

H&E staining is a foundational technique in histopathology that provides a morphological overview of tissue architecture by differentially staining nuclei and cytoplasmic components [14, 15]. In the context of immune infiltration, H&E enables qualitative and semiquantitative assessment of TILs based on their morphology. TILs have been found to be a prognostic biomarker across various cancer types, including triple-negative breast cancer (TNBC), epidermal growth factor receptor 2 (HER2+) breast cancer [16, 17] and non-small cell lung cancer [18–20]. Breast cancer has emerged as a leading model for standardized TIL evaluation using H&E because of its clinical relevance of immune infiltration in predicting treatment response [21–24]. Early studies assessed both intratumoral and stromal TILs (sTILs) and demonstrated sTILs are more reproducible across studies [25–27]. Salgado and colleagues [21] proposed estimating sTILs as the proportion of stromal area occupied by lymphocytes, leading to the development of international guidelines that standardized the scoring protocol. Subsequent multicentre ring studies demonstrated its reproducibility and clinical relevance, supporting its adoption in diagnostic workflows [9]. Beyond breast cancer, H&E-based TILs assessment has also been applied to other cancer types. For example, sTILs is also used in non-small cell lung cancer [28], and the guidelines proposed by the International TILs Working Group have been adapted for colorectal cancer (CRC) [29]. Nevertheless, H&E-based assessment only provides a general view of TILs density and does not differentiate between immune cell subtypes, which limits our understanding of the function of different immune cell types in cancer progression [30]. This highlights the need for complementary approaches such as IHC for deeper immune profiling [31].

#### Immunohistochemistry staining

IHC is a widely used technique that enables the visualization of specific cellular components within tissue sections through antigen-antibody interactions [32]. By targeting cell surface or intracellular markers with specific antibodies, IHC allows for the identification and quantification of distinct immune cell subsets, providing a more detailed characterization of the tumour immune microenvironment compared to conventional H&E staining. One of the most prominent examples of IHC-based immune profiling is the development of the Immunoscore in CRC [33–35]. By quantifying CD3+ and CD8+ T cells within the tumour core and invasive margin, Immunoscore provides a standardized, reproducible metric that has demonstrated strong prognostic significance across multiple independent cohorts [36, 37]. Studies have shown that Immunoscore outperforms traditional TNM (tumour, node, and metastasis) staging in predicting patient outcomes, underscoring the clinical relevance of incorporating immune parameters into cancer classification systems [35, 38]. The prognostic value of Immunoscore was assessed in other cancer types [39, 40], more cohorts are needed for a comprehensive evaluation. Despite the prognostic power of Immunoscore, it has limitations. Traditional IHC can only stain one marker per tissue section, lacking the capacity for comprehensive analysis for tumour immune contexture. Manual scoring by pathologists can be labour-intensive and subject to interobserver variability, resulting in inconsistent results among pathologists.

## AI-based digital pathology approaches

Digital pathology refers to the process of digitizing conventional histopathology slides (typically H&E or IHC stained) using whole-slide scanners producing what is commonly known as whole slide images (WSIs). Significant advances in image resolution, computing power, and storage infrastructure over the past two decades have transformed digital pathology into an active research and diagnostic field [11]. Digital pathology enables automated quantification of TILs and other biomarkers using digital image analysis (DIA). Multiple commercial and open source systems have been developed for extracting meaningful information from histopathology images, including HALO (https://indicalab.com/halo/), Visiopharm (https://visiopharm.com/), QuPath [41], CRS4 Digital Pathology Platform [42] etc., facilitating improved annotation and prediction tasks beyond manual assessment for cancer research. For example, Stålhammar *et al*. found that DIA-based breast cancer subtype classification outperformed manual biomarker assessment [43]. McIntire and colleagues observed that DIA outperformed manual biomarker scoring for Luminal B subtype classification of breast cancer [44]. Other studies [44–47] also showed that DIA can quantify TILs in breast cancer and reproduce clinical findings by pathologists. The advantage of using DIA in tumour assessment is that it has potential in reducing uncertainty and variability introduced by pathologists’ manual assessment [41, 48].

Despite the advantages of commercial DIA, it lacks the capacity of customizing AI models and analysing large datasets. Researchers have focused on developing more customized and flexible AI models to score TILs from H&E images via different tasks such as image segmentation, feature extraction, and lymphocytes classification [49–53]. For instance, a morphological-based support vector machine (SVM) classifier was trained to detect tumour, lymphocytes, and stromal cells in breast cancer, revealing that higher number of TILs is associated with better outcome for a subset of estrogen receptor-negative (ER-) patients [53]. Saltz *et al*. combined a segmentation convolutional neural network (CNN) and a classification CNN to identify lymphocyte-infiltrated regions in H&E using TCGA data, which successfully characterized differential TILs patterns for 13 cancer types and survival outcome [54]. An integrated joint region- and cell-level semantic segmentation model was applied to WSI to quantify sTILs in TNBC patients showed high consistency with pathologists’ consensus [55]. Other automated pipelines that used region annotation and cell segmentation such as Fast TILs [56] and TILScout [57] also showed the prognostic value of TILs in various cancer types. However, these pipelines rely on heavily detailed annotation by expert pathologists. More recently, the AI foundation model such as UNI has been developed and evaluated on various tasks including TILs detection, enabling data-efficient and transferable feature representations [58]. Instead of segmentation or cell annotation methods, two-stage label-efficient deep learning pipelines have been developed using features derived from foundation models followed by training a multiple instance learning model, achieving high concordance with expert pathologists [59, 60]. Collectively, these AI-based tools paved the way of automatically characterizing tumour-immune microenvironment in a more reproducible and scalable manner.

Despite significant advances, computational pathology still faces notable limitations in assessing tumour immune infiltration [61]. Scalability remains a practical challenge, as deep learning models typically require substantial computational resources, particularly during model training, which may limit their application to large-scale datasets. In addition, most methods rely on conventional staining techniques, such as H&E or IHC, which provide limited molecular depth. Batch effects arising from differences in acquisition sites, staining protocols, and image scanning can introduce systematic bias in feature extraction when applying deep learning models to histopathology images [62, 63]. As a result, irrelevant features could be learned which impacts model generalizability [64]. To mitigate these limitations, a range of strategies have been proposed, including stain normalization and data augmentation to improve robustness across institutions [65, 66], reflecting ongoing efforts to improve the reliability of computational pathology.

## Molecular-based estimation

### Bulk platform

While histology provides valuable insight into tumour morphology, it lacks molecular markers that can elucidate cancer progression and inform therapeutic strategies. RNA plays a key role in regulating cellular processes in the development of cancer. Bulk RNA sequencing provides a high-throughput and cost-effective approach to profiling the global transcriptomic landscape of the entire sample. However, as bulk RNA-seq captures the average gene expression across all cells within a sample, it inherently mixes signals from diverse cell types in tissues, which cannot capture the heterogeneity of cell types in TME [67]. Computational deconvolution methods have been developed to extract information of cell-type specific expressions from these mixed profiles.

Many deconvolution algorithms have been developed to estimate the proportion or abundance of distinct cell populations within bulk samples based on their gene expression signatures. Im and Kim reviewed RNA deconvolution methods and their applications in characterizing tumour microenvironment [68]. Examples include: CIBERSORT [69], which is a deconvolution method applied to microarray or RNA-seq data that uses a reference immune gene signatures to estimate the fraction of cell subsets from bulk tissue, showing high sensitivity and specificity when applied to simulated solid tumours with varying immune infiltration. TIMER [70,71], which applied a constrained linear regression model to deconvolve gene expression and facilitated evaluation of clinical impact of different immune cell types. Other deconvolution methods [72–75] also suggested their clinical potentials in estimating immune infiltration and its associations with survival outcome.

### Advances from bulk platform (single-cell RNA-seq and spot-based spatial transcriptomics)

In contrast to bulk RNA-seq, which measures average gene expression across heterogeneous cell populations, recent advances have moved towards single-cell RNA-seq, enabling quantification of gene expression at single-cell resolution and, more recently, in a spatially resolved manner.

An early scRNA-seq analysis of breast cancer patients clearly separated tumour cells and tumour infiltrating immune cells using a single-cell marker free approach and identified diverse tumour infiltrating immune cell types that indicate tumour progression and metastasis [76]. A pan-cancer single-cell landscape of T cells revealed two groups of tumour infiltrating T cell compositions (terminal exhausted CD8+ T cells versus tissue-resident memory CD8+ T cells) were associated with clinical outcome [77].

While recent advances offer single-cell resolution, they typically lack the spatial information. As a result, the field faces a trade-off between high-resolution single-cell approaches and spot-based technologies that preserve spatial context. sST quantifies gene expression at spot level with each spot typically containing greater than 10 cells [78–80], which again measure a mixture of cell signals. Thus, deconvolution methods are required to infer cell-type composition at each location. A recent review on spatial deconvolution methods [81] summarized a variety of approaches, including regression, Bayesian, ensemble algorithm, and deep-learning-based frameworks. The inferred cell-type composition with spatial information allows characterizing tumour immune microenvironment in a spatially resolved manner. For example, applying SpatialDecon to a non-small cell lung cancer dataset uncovered seven tumour immune microenvironment patterns corresponding to distinct regions and also transitioning boundaries in the tumour [82–85].

While deconvolution methods have significantly advanced our ability to study tumour-immune interactions without the need for single-cell resolution, there are rooms for further progress. Tumor Deconvolution DREAM Challenge consortium benchmarked a large number of deconvolution methods on inferring immune infiltration level, suggesting the challenges in predicting minor lineages and functional T cell states [86]. With refined marker genes, advanced computational methods can be developed to address these challenges. Compared to bulk RNA-seq deconvolution, spatial deconvolution methods often leverage H&E images and spatial neighbourhood, which has the potential to capture immune infiltration patterns in tumour immune microenvironments that may indicate prognostic outcome and disease subtypes. However, there is no comprehensive evaluation on how these methods perform for identifying tumour immune infiltration patterns that are robustly related to outcome.

### Single-cell resolution spatial platform

Since the advent of multiplexed imaging approaches in the past decade, imaging-based spatial technologies have evolved to complement digital imaging microscopy with molecular profiling, providing single-cell or even subcellular resolution of gene and protein expression. Fluorescence *in situ* hybridization (FISH)-based methods [87], such as MERFISH [88], seqFISH+ [89], and CosMx [90], and *in situ* sequencing-based method such as Xenium allow for a direct characterization of immune infiltration with spatially resolved single-cell analysis. A wide range of integrated toolkits support these analyses by implementing a variety of spatial statistics using these spatial omics data, including SPIAT [91], Voyager [92] , scFeatures [93], spicyR 94], and Squidpy [95].

There are a number of metrics previously used on digitalized histological samples with automated cell type identification to characterize TME [96]. These metrics can be broadly classified into (i) density-based metrics which examine cell abundance/cell type proportion within a given area and (ii) higher order spatial pattern metrics which summarize spatial clustering and cell type associations. Density-based metrics can also be called first-order metrics, quantifying immune cell abundance (e.g. CD8+ T cells per mm^2^) which provides a measure of overall infiltration. They are simple to compute and interpret but do not capture spatial relationships. In contrast, higher order spatial metrics assess how immune cells are spatially distributed in TME and interact with other cell types or immune subsets, revealing patterns such as clustering at the invasive margin or exclusion from the tumour core. They either treat each cell as a point or use its expression value as input. These statistics include point process statistics such as Ripley’s *K* function or spatial autocorrelation metrics such as Moran’s *I* and Getis-Ord statistics. We divide various spatial statistics into first order (density-based metrics) and second order (spatial correlation metrics) statistics and describe their calculations in Table 1.

**Table 1:** First order and second order spatial statistics for quantifying immune infiltration level or pattern.

**Spatial measures**	**Formula**	**Notes**
First order (How the intensity or value of a variable varies across space)		
Cell density	$\frac{N_{cells}}{Area}$	Count of cells, normalized for area. TILs density is calculated as the number of TILs/tissue area.
Kernel density	$f(x)=\frac{\sum_{i=1}^nk\left(x-{x}_i\right)}{h}$	Kernel smoothing to estimate density. Here is the uniformly corrected version, where $h$ is bandwidth, ${x}_i$ is the coordinate of the $i$th point.
Localized entropy	$Entropy=-{\sum}_{i=1}^np\left({x}_i\right){\mathit{\log}}_2p\left({x}_i\right)$	Localized entropy measures the diversity of cell types in local regions. $p\left({x}_i\right)$ is the proportion of cell type $i$ in a local region. Localized entropies can be summarized to represent spatial heterogeneity [91].
Second order (Spatial dependence or correlation between observations at different locations)		
Cross-type density ratio	$r\left(x,y\right)=\frac{c_1\left(x,y\right)}{c_2\left(x,y\right)}$	${c}_1\left(x,y\right)\ and\ {c}_2\left(x,y\right)$ are the density of two cell types.
Density variance	$Var(\hat{\lambda})=\frac{1}{\left|A\right|}{\int}_A(\hat{\lambda}(x)-\mathrm{\lambda} ) dx$	The degree of variability in the density of a cell type within an image. $\hat{\lambda}(x)$ is the estimated density at location $x$. $\mid A\mid$ is the area of the image.
Global Moran’s *I*	$I=\frac{n\sum_i{\sum}_j{w}_{ij}\left({Y}_i-\mathrm{Y}\right)\left({Y}_j-\mathrm{Y}\right)}{\left({\sum}_i{\sum}_j{w}_{ij}\right){\sum}_i\left({Y}_i-\mathrm{Y}\right)}$	The extent to which a variable is correlated with itself through space. The variable could be cell densities, gene expression level, etc. $n$ is the total number of cells/spots, ${Y}_i$ is the observed value of the variable at location $i$, $Y$ is the mean of all ${Y}_i$, ${w}_{ij}$ is spatial weight between location 𝑖 and 𝑗.
Ripley’s *K [97]*	$\hat{K}(r)=\frac{\left|W\right|}{n\left(n-1\right)}{\sum}_{i=1}^n{\sum}_{j=1,j\ne i}^n1\{{d}_{ij}\le r\}{e}_{ij}(r)$	Spatial correlation in point pattern. It can quantify whether two cell types are clustered or dispersed. (details see [97])
Kontextual [98]	$\hat{K_{ab}^{context}}(r)=\frac{1}{\sum_{x_i\in{X}^a}{\lambda}_{context}{x}_i}$ ${\sum}_{x_{i\in{X}^a}}{\lambda}_{context}{x}_i$ ${\sum}_{x_{j\in{X}^b}}\frac{1\left({d}_{ij}\le r\right)}{\lambda_{context}{x}_j\frac{n_b}{n_{context}}}e({x}_i,{x}_j,r)$	A modified inhomogeneous multitype *K*-function, accounting for tissue heterogeneity and region selection. (details see [98])
Average pairwise distance between two cell types [91]	${APD}_{AB}=\frac{1}{n_A\cdot{n}_B}{\sum}_{i=1}^{n_A}{\sum}_{j=1}^{n_B}d({a}_i,{b}_j)$	Cell type colocalization pattern. ${n}_A$, ${n}_B$ are the numbers of cells of cell type A and B. $d({a}_i$, ${b}_j)$ is the distance between cell $i$ in cell type A and cell $j$ in cell type B.
Average minimum distance between cells [91]	${AMD}_{AB}=\frac{1}{n_A}{\sum}_{i=1}^{n_A}{\mathit{\min}}_jd\left({a}_i,{b}_j\right)$	Cell type colocalization pattern. ${n}_A$ is the number of cells of cell type A. $d({a}_i$, ${b}_j)$ is the distance between cell $i$ in cell type A and cell $j$ in cell type B.
Mixing score [99]	$Mixing\ score=\frac{n_{ref\ to\ target\ interactions}}{n_{ref\ to\ ref\ interactions}}$	${n}_{ref\ to\ target\ interactions}$ is the number of interactions between reference cells and target cells. ${n}_{ref\ to\ ref\ interactions}$ is the number of interactions between reference cell types.
Getis-Ord general G-statistic	$G=\frac{\sum_{i=1}^n{\sum}_{j=1}^n{w}_{ij}{x}_i{x}_j}{\sum_{i=1}^n{\sum}_{j=1}^n{x}_i{x}_j}$	Hotspots and coldspots analysis. *n* is the number of cells/spots, ${x}_i$, ${x}_j$ are values of the variable at location $i$ and $j$. ${w}_{ij}$ is the spatial weight between location $i$ and $j$.

**Table 2:** Summary of advantages and disadvantages of different platforms for estimating immune infiltration level.

	Traditional method (H&E/IHC)	AI-based digital pathology	Transcriptomic (bulk)	Spatial omics (sST, imaging-based spatial omics)
Cell type resolution	H&E: Can only identify general lymphocytes. IHC: With immune markers, can identify major immune cell types (e.g. CD4, CD8 B cell).	Can only identify general lymphocytes.	Quantifies gene expression across mixed population of cells. Cannot identify specific cell types. Need to perform cell-type deconvolution analysis to infer cell-type specific gene expression.	sST: Provides a mixture of cell types in each spot. Need to perform spatial cell-type deconvolution analysis to infer cell-type specific gene expression for each spot. Imaging-based spatial omics: Can identify specific cell type and cell state by cell segmentation and cell phenotyping based on markers, enabling downstream analysis characterizing immune infiltration.
Spatial context	H&E: Have spatial information that informs tissue morphology. Experienced pathologists can select ROIs (e.g. stromal, intratumoral region) to estimate TILs. IHC: Same as H&E. With cell-type marker information, infiltration of specific immune cell types is allowed to be assessed.	Provides spatial context based on cell locations. With deep learning-based cell identification, TILs can be calculated automatically at tile level and aggregated to WSI level. TILs can also be calculated within ROIs.	No spatial context.	Both provide spatial context with various resolution levels.
Clinical interpretability	H&E and IHC: Widely used in clinical settings, easy to interpret as the stains inform cell and tissue structures associated with disease that can be easily visualized.	Depending on the approach that an AI model adopts, these AI tools derived TILs are not always highly interpretable. For example, cell segmentation-based TILs estimation is more interpretable compared to patch-based classification [100].	Hard to interpret for clinicians. Various deconvolution methods exist with no standardized pipeline and report.	Has the potential in clinical settings as it provides spatial information. However, there is no standard pipeline for data generated from different platforms. It is hard to interpret for clinicians without a bioinformatics background [101].
Scalable to large cohorts	Cheap but labour intensive.	Although these AI driven tools are scalable compared to traditional methods, their scalability across cohorts is constrained by factors such as computational requirements, stain variability across laboratories, and differences in tissue preparation protocols [62, 102].	Although bulk RNA-seq is cost-effective compared to single-cell and spatial technologies, currently it is mainly used for research due to the complexity of cell type deconvolution [86].	Expensive, only for research settings.
Performance assessment/validation strategy	They follow internationally established guidelines. Often validated by inter-observer variability (concordance of TILs score assessed by different pathologists) [21].	AI-based methods use task-specific metrics such as sensitivity, specificity, F1 score. These metrics are sensitive to training data, which are often manually annotated by clinicians.	Methods evaluation often uses simulated and real data to calculate metrics such as RMSE.	sST: Similar to bulk transcriptomic, deconvolution methods are validated using both simulated and real data. Metrics such as RMSE and JSD are used for evaluation. The agreement between predicted results and biological spatial organization are usually assessed using real data.

Except for conventional spatial metrics used in ecology research, recent efforts have been made in developing advanced metrics/tools to dissect healthy tissue structure and tumour microenvironment and applying these to data generated by new spatial technologies. These newly developed methods have focused on quantifying cell-type associations to answer different biological questions. For example, *Spatiopath* generalizes Ripley’s *K* function to deal with both cell-cell and cell-structure interactions [103], identifying spatial associations between mast cells and T cells and the tumor epithelium. *Kontextual*, a method extends homogeneous multitype *K*-function to inhomogeneous multitype *K*-function, enabling elucidating subtle spatial relationships in different contexts which could not be identified by using the original *L*-function [98].

**Figure 2:**
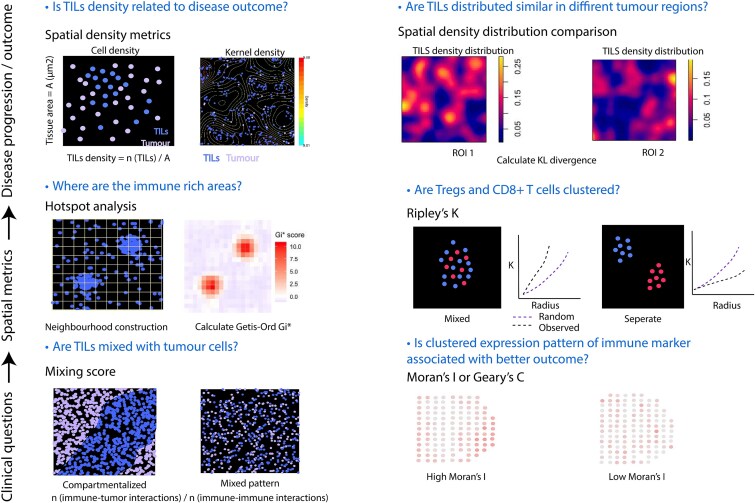
Spatial metrics addressing different clinical/biological questions in immune infiltration analysis. Representative spatial statistics used to quantify tumour immune microenvironment at different analytical levels are presented with different clinical questions. These spatial statistics are often tested against disease progression or outcome. First-order density-based metrics (e.g. cell density, kernel density) quantify immune cell abundance and distribution within defined ROI and can be used to test whether TIL density associates with disease outcome. TILs densities distribution from two ROIs and be compared using KL divergence. Second-order spatial correlation metrics assess spatial dependency between cells or molecular features, such as hotspot analysis for immune-enriched regions, mixing scores for tumour-immune proximity, Ripley’s *K* function for spatial clustering of immune subsets, and Moran’s *I* or Geary’s *C* for spatial autocorrelation of molecular expression. Together, these metrics form a hierarchical framework: from density estimation to higher-order spatial association analysis, guiding the interpretation of the spatial organization of TILs.

Although many spatial metrics have been applied to imaging-based spatial omics data to capture spatial patterns, caution is warranted when choosing spatial statistics to answer certain questions. Summers *et al*. summarized the application of spatial metrics to decipher cellular relationships and tissue structure and concluded that awareness should be raised on which spatial statistics is appropriate for a given image [104]. Emons *et al*. discussed the advantages, challenges, and nuances of various spatial statistics when applied to different modalities of spatially resolved omics data, and concluded that choosing spatial analytical approach depends not only on modalities but also on research questions [105]. Figure 2 summarizes representative spatial metrics that can be applied to address distinct clinical or biological questions regarding immune infiltration, ranging from first-order density measures to second-order spatial association analyses.

## Multimodal data integration

It is widely recognized that different data modalities provide complementary insights, and integrating them is essential to capture the full complexity of the tumour microenvironment. For instance, H&E images offer rich morphological information but lack molecular resolution. Conversely, molecular-based platforms such as Visium often rely on H&E to anchor gene expression data to specific cells and tissue structures, facilitating data interpretation by providing spatial context. With the rapid development of computational integration methods, multimodal data can now be combined to yield a more comprehensive understanding of the tumour immune microenvironment, enabling the derivation of more accurate and robust features for patient stratification and outcome prediction.

**Figure 3:**
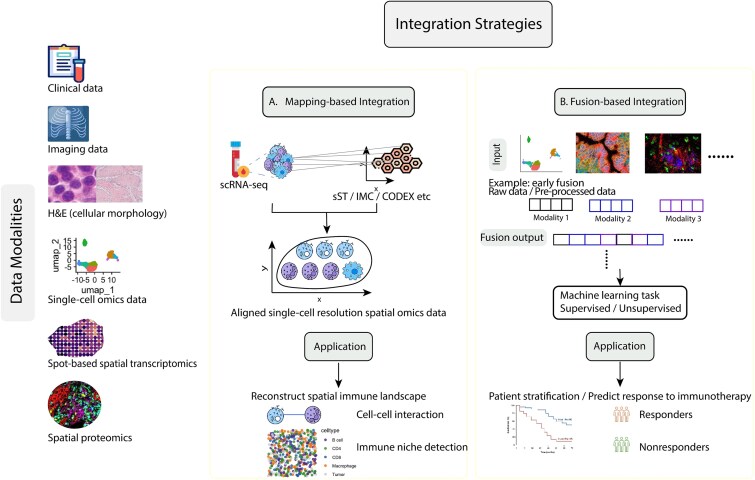
Overview of data integration strategies using spatial and multimodal omics data. Different data modalities, including clinical, imaging, histological (H&E), single-cell omics, spot-based spatial transcriptomics, and spatial proteomics, provide complementary biological information. Two major integration strategies are illustrated. (A) Mapping-based integration: scRNA-seq data can be mapped onto spot-based spatial transcriptomics (sST) or proteomics data to reconstruct single-cell resolution spatial maps, enabling analyses such as spatial immune landscape reconstruction, cel-cell interaction, and immune niche detection. (B) Fusion-based integration: multiple modalities (e.g. molecular, imaging, and clinical data) are jointly modeled through feature fusion or joint representation learning (using early fusion as an example strategy), often within supervised or unsupervised machine learning tasks. This approach facilitates applications such as patient stratification and prediction of immunotherapy response.

Data integration strategies can be generally divided into data mapping methods and data fusion methods (Fig. 3). Data mapping methods typically integrate data by aligning one modality data to other modalities (Fig. 3A). For example, Tangram is a deep learning method that maps sc/snRNA-seq data onto various types of spatial omics data derived from the same tissue, enabling the construction of genome-wide spatial maps at single-cell resolution [106]. STvEA can annotate cell types and states that cannot be identified by antibody panels of highly multiplexed cytometry data by mapping scRNA-seq to spatial omics data [107]. Using STvEA, Quek *et al*. integrated CITE-seq and CODEX data from melanoma patients to perform comprehensive analysis of response and resistance to immunotherapy in spatially resolved manner [108]. The results demonstrated T cell proportion is not a prognostic indicator without spatial resolution. These methods will offer new opportunities to reconstruct the spatial landscape of the tumour immune microenvironment.

The other popular strategy is data fusion methods, which take different modalities of data as inputs and learn a joint representation that integrates complementary information across data types (Fig. 3B). Depending on the state of the input and which information across modalities is combined, data fusion strategies can be broadly categorized into early, intermediate, and late fusion approaches. Early fusion integrates modalities at the feature level without unimodal representations learned. It often learns the relationship across modalities from low-level features. Instead of using low-level features as input, intermediate fusion first extracts features from each modality and combines these unimodal representations through fusion approaches such as concatenation, possibly followed by attention-based weighting [109]. Late fusion, by contrast, aggregates modality-specific predictions or decisions. Data fusion has been widely adopted in cancer research to integrate clinical, imaging, and omics data for the prediction of treatment response, thereby guiding treatment decision-making in clinical settings [110–114]. More recently, advanced computational data fusion frameworks have been developed to learn joint representations that link molecular features from single-cell data with their spatial context, enabling integrative analysis across omics modalities. For example, as an early fusion, model-free approach, MaxFuse integrates different modalities by linking correlated features across datasets, allowing for effective integration under weak cross-modality correlations [115]. The authors demonstrated its capacity to link three modalities (CODEX-snRNA-snATAC), enabling the mapping of spatial enrichment of transcription factor binding site accessibility. Unlike model-free alignment approaches, CellLENS represents an intermediate fusion strategy based on deep learning architecture, focusing on delineating immune cell populations and associated cell states, uncovering distinct tumour infiltration patterns and predicting clinical outcomes [116]. CellLENS adopts a hybrid deep learning architecture combining CNNs to extract image features that capture local spatial patterns, parallel spatial and expression graph neural networks (GNNs) connected by multilayer perceptrons (MLPs) for cross-modal feature integration. This design enables downstream tasks such as cell-type identification and spatial pattern analysis. Collectively, data fusion approaches leveraging multimodal datasets generated by advanced technologies open new avenues for predicting therapeutic responses and characterizing the tumour immune microenvironment.

## Comparative discussion

### Comparison of spatial resolution across platforms

Spatial resolution is one of the most critical factors in estimating immune infiltration. Different platforms offer various resolutions for quantification while each of them has advantages and limitations. In clinical settings, pathologists choose regions of interest (ROI) to estimate TILs. For example, for breast cancer, stroma-riched regions are usually chosen for visual TILs quantification. This cost-effective conventional assessment is used in the current clinical routine of TILs estimation. However, assessing TILs in a focal area can generate substantial inter- and intra-observer variability due to subjective interpretation of cell morphology and tissue components. Van Bockstal *et al*. evaluated the consistency of TILs estimation across pathologists and found acceptable correlation for sTILs at group level but inconsistent estimation for individual patients [117]. Variability is also dependent on tissue regions selected for TIL estimation. For example, stromal TILs interobserver variability is lower than intratumoral variability [118]. AI-based digital pathology improved reproducibility by automatically classifying major cell types. However, estimating TILs at WSI level is difficult because of the large size of WSI, which cannot be handled by deep learning algorithms with limited computational resources [119]. Splitting WSI into smaller patches and estimating TILs for each patch is a common strategy. Patch level features are usually aggregated using algorithms or summary statistics to generate WSI infiltrating estimation. This approach is often determined by how aggregation is performed on patch-level results.

For molecular-based platforms with spatial information such as 10x Visium, which aggregates signals over 50-100 μm in a spot that contains multiple cell types, cell type deconvolution is needed. The inferred cell type distribution can be affected by reference data and algorithms. Wang and colleagues simulated immune infiltrating spots and benchmarked the performance of various deconvolution algorithms and found they performed differently on detecting immune infiltrating spots [120]. Therefore, these could affect the estimation of TILs. Imaging-based ST technologies, such as CODEX and MIBI, recover subcellular localization of immune markers, offering high resolution at the cost of limited marker panels. These allow for downstream single-cell-level spatial analysis to assess immune infiltration level. Although these technologies have achieved single-cell and even subcellular-level resolution, they focus on a small area of a whole tissue, which may overestimate or underestimate the true infiltration level at the whole tumour level.

### What is the immune cell type resolution of each platform?

Distinguishing immune cell types is essential for understanding their functionality and responses to immunotherapy. Different immune cell types and cell states within TME play distinct roles at different tumour stages [121]. Routine H&E and AI-based digital pathology assess TILs based on H&E images, which only provide morphological context without cell surface markers. Thus, specific immune cell types cannot be detected. IHC, which is designed to identify cell types by staining immunopositive cells, can distinguish B cells (CD20+) and T cells (CD3+ and CD8+) by certain cell markers. However, IHC-based approaches offer limited cell type resolution, often relying on a small number of markers that constrain the ability to distinguish functionally diverse immune cell subsets within the tumour microenvironment. For bulk RNA-seq (mixture of thousands to millions of cells) and spot-based spatial transcriptomics (mixture of several cells per spot), cell type abundance needs to be inferred using deconvolution methods. The deconvolved cell type resolution varies substantially, depending on the granularity of the reference dataset and the algorithm’s ability to resolve closely related immune subtypes, e.g. distinguishing CD8+ T cells from exhausted CD8+ T cells or macrophages into M1 and M2 phenotypes. Imaging-based spatial omics data enables high-resolution cell type annotation, benefiting from spatial context and direct molecular detection, but remains constrained by a limited number of markers and dependency on robust panel design for fine-grained immune cell subtype resolution. In addition, immune cell phenotypes can be different depending on tissue context, which makes it challenging to perform accurate cell type classification [122].

### Can spatial immune organization be constructed?

Assessing TILs at the whole tumour level is not able to inform local spatial patterns which reflect the dynamic interactions between immune cells and tumour microenvironment. Evidence showed that tumours that have similar sized immune infiltrates exhibit distinct spatial organizations, which are associated with survival outcome [99], highlighting the importance of constructing clinically meaningful spatial patterns.

One key structure is the cellular niche, also refers to the spatial domain, which represents a cluster of cells interacting within a microenvironment. Such cell communities can be derived using a variety of assays. For example, advanced computational methods applied to H&E and tissue imaging data can detect immune niches, which have been shown to improve patient risk stratification in lung cancer under different therapeutic strategies [123]. Recent spatial omics technologies allow molecularly resolved reconstruction of the tumour immune landscape at a higher resolution. Typically, this involves an initial step of spatial deconvolution or cell segmentation, followed by computational modelling to characterize immune niches [124]. A growing number of methods have been developed to identify cell niches using data generated by different spatial omics technologies [125–128]. For instance, CellCharter applied to lung cancer cohorts identified tumour-associated neutrophil infiltration indicating the activation of a response-to-hypoxia state, associated with poor prognosis [126]. These methods offer unprecedented opportunities to uncover patient-, condition-, and disease-specific cell niches that could serve prognostic markers in the clinic.

However, most methods identify spatial cell clusters without clear biological interpretations due to limited ground truth, restricted marker panels, and insufficient functional validation. Future work should aim to bridge computationally defined spatial clusters with immunological mechanisms and clinical outcomes to achieve interpretable, prognostically relevant spatial immune organization.

### Comparison of clinical interpretability

Classical histopathology assessment, such as evaluating stromal TILs in breast cancer, is well-standardized and reproducible, with established international guidelines and demonstrated prognostic value, making it highly interpretable in clinical settings [21,129]. IHC adds moderate resolution by identifying specific immune cell subsets (e.g. CD3^+^ T cells), and clinical scoring systems such as the Immunoscore in CRC have shown strong prognostic utility and robustness across centers [33, 35].

While classical histopathological and immunohistochemical assessments provide clinically interpretable and reproducible measures of immune infiltration, they are limited by their coarse spatial resolution and lack of molecular depth. Spatial omics technologies link molecular data to tissue architecture, offering unprecedented opportunities to derive quantitative spatial biomarkers that can inform disease outcome. Tumour-immune infiltration patterns can be captured by calculating spatial metrics tailored to specific questions (Fig. 2). Studies have shown that immune hot/cold and compartmentalization revealed by spatial omics data can be linked to survival outcome [99,130]. Moreover, immune niche or cell community detection has identified distinct immune cell clusters showing different characteristics that differ between conditions [125]. Moving forward, aligning these spatial discoveries with clinical pathology research and underlying immunological mechanisms will be essential for developing clinically actionable spatial biomarkers [131].

Despite the promise of computational pathology for TILs quantification, the limited interpretability of deep learning models poses a challenge for their clinical translation. For example, cell segmentation-based approaches for TILs estimation are considered more interpretable, as they provide cell-level classification on WSI that can be visually inspected by pathologists. In contrast, patch-based classifications are less intuitive for clinical interpretation [100]. To address these limitations, *post hoc* interpretability approaches, such as attention maps and class activation maps, have therefore been increasingly adopted to highlight image regions contributing to model predictions, partially improving interpretability by producing heatmaps that can be visualized by clinicians [132, 133].

Similarly, despite their enhanced molecular resolution and capacity to reveal mechanistic insights, spatial omics technologies face substantial challenges in clinical translation. Variability in tissue sampling, platform-specific biases, and limited consensus on data analysis pipelines substantially affect reproducibility across studies [134]. Consequently, they remain a largely research platform where discovery results will translate into a more ‘accessible’ platform to guide clinical decision-making. Moving forward, balancing biological insight with clinical feasibility and standardization is essential for successful translation into patient care.

### Comparison of performance assessment and evaluation strategies across platforms

A key challenge in comparing immune infiltration quantification methods across platforms lies in how ‘performance’ is defined and evaluated. Estimates of immune infiltration are inherently indirect and depend on the modality or assay used for inference, each of which adopts different evaluation strategies. Consequently, performance assessment is platform- and task-dependent, making it challenging to benchmark across platforms. Here, we summarize the evaluation strategies commonly adopted for different platforms.

Classical histopathological assessment follows internationally established guidelines to standardize TILs quantification by pathologists [21,129]. Performance is typically evaluated through inter-observer reproducibility and associations with clinical outcomes, including survival or treatment response. Importantly, as noted by the TILs Working Group, such evaluations primarily assess concordance rather than accuracy against ‘ground truth.’ While this is acceptable in clinical settings, the lack of ground truth poses challenges for the validation of automated methods [135]. Validation of AI-based digital pathology approaches often rely on task-specific metrics such as sensitivity, specificity, F1 score, or Dice coefficient. These metrics are commonly used in the context of predicting TILs presence/absence, density, or clinical outcomes [136,137]. However, these metrics are sensitive to training data, which are derived from manual annotations by pathologists. As a result, the concordance between model prediction and pathologist score may not necessarily equate to biological accuracy or clinical validity [135,138].

For transcriptomics-based platforms, including bulk RNA-seq and sST, the evaluation of cell-type deconvolution methods relies on both simulated and real datasets. Performance is assessed by comparing estimated cell proportions against known ground truth in simulations or against expected biological patterns and tissue structures in real experimental data [139,140]. Commonly used accuracy metrics include mean absolute error (MAE), root mean squared error (RMSE), Jensen–Shannon divergence (JSD). Except for accuracy, recent benchmarking studies also emphasize robustness, consistency, and stability when assessing performance[140, 141]. More recently, a benchmark study extended the evaluation to different transcriptomic platforms, including bulk transcriptomics and spatial transcriptomics using different technologies [142]. This illustrates how cross-platform comparability can be assessed despite technological differences. Such evaluation strategies offer a practical reference for future performance assessment of immune infiltration estimation methods across diverse transcriptomic platforms.

In summary, evaluation should focus on whether a method’s validation strategy is appropriate for the specific biological and clinical question being addressed. This perspective underscores the importance of task-aware and modality-aware interpretation of performance.

## Conclusion

Tumor immune infiltration is a complex and spatially heterogeneous process. Estimating TILs and characterizing tumour immune microenvironment are critical for understanding immune responses and guiding therapy. However, multiple platforms can be used to quantify TILs or tumour infiltration level at different resolutions, and each of them has some advantages and disadvantages. In this review, we have compared a wide range of methodological frameworks across histological, bulk RNA-seq and the emerging spatial omics platforms. Each platform brings unique strengths and limitations in terms of resolution, interpretability, and clinical utility. Rather than identifying a single best approach, our comparative synthesis highlights the importance of platform-aware method selection and integrative strategies. Future advances will likely emerge from combining spatial, molecular, and clinical data within robust statistical frameworks and pipelines to enable more informative and actionable immune profiling in cancer.

**Key Points**

- This review provides a comprehensive overview on experimental platforms and computational approaches for quantifying tumour immune infiltration level, spanning classical histology, bulk and spatial transcriptomics, and multiplexed imaging data.
- Immune infiltration can be estimated across different data resolutions, from proportion-based deconvolution methods in bulk RNA-seq to spatially informed metrics derived from spatial omics technologies.
- We highlight recent advances in computational methods for multimodal data integration and their potential to unify multimodality data for characterizing immune infiltration patterns.
- Comparative discussion provides insights into the strengths, limitations, and clinical applicability of current advanced technologies.

## Data Availability

No data was generated or analysed in this review.

## Notes

The authors are from Sydney Precision Data Science Centre at the University of Sydney. Their expertise includes statistical bioinformatics, especially method development in single-cell and spatial omics.
